# Emerging Radiotherapy Technologies for Head and Neck Squamous Cell Carcinoma: Challenges and Opportunities in the Era of Immunotherapy

**DOI:** 10.3390/cancers16244150

**Published:** 2024-12-12

**Authors:** Carmen Kut, Harry Quon, Xuguang Scott Chen

**Affiliations:** 1 Department of Radiation Oncology and Molecular Radiation Sciences, Johns Hopkins University, Baltimore, MD 21287, USA; ckut1@jhmi.edu (C.K.); hquon2@jhmi.edu (H.Q.); 2 Department of Radiation Oncology, University of North Carolina, Chapel Hill, NC 27599, USA

**Keywords:** head and neck cancer, intensity modulated radiotherapy, chemoradiotherapy, proton therapy, stereotactic body radiotherapy, spatially fractionated radiotherapy, immunotherapy

## Abstract

Radiotherapy (RT) is an integral component in the multidisciplinary management of patients with head and neck squamous cell carcinoma (HNSCC). Significant advances have been made toward optimizing tumor control and toxicity profiles of RT for HNSCC in the past two decades. The development of intensity modulated radiotherapy (IMRT) and concurrent chemotherapy established the standard of care for most patients with locally advanced HNSCC around the turn of the century. More recently, selective dose escalation to the most radioresistant part of tumor and avoidance of the most critical substructures of organs at risk, often guided by functional imaging, allowed even further improvement in the therapeutic ratio of IMRT. Other highly conformal RT modalities, including intensity modulated proton therapy (IMPT) and stereotactic body radiotherapy (SBRT) are being increasingly utilized, although there are gaps in our understanding of the normal tissue complication probabilities and their relative biological effectiveness. There is renewed interest in spatially fractionated radiotherapy (SFRT), such as GRID and LATTICE radiotherapy, in both palliative and definitive settings. The emergence of immune checkpoint inhibitors (ICIs) has revolutionized the treatment of patients with recurrent and metastatic HNSCC. Novel RT modalities, including IMPT, SBRT, and SFRT, have the potential to reduce lymphopenia and immune suppression, stimulate anti-tumor immunity, and synergize with ICIs. The next frontier in the treatment of HNSCC may lie in the exploration of combined modality treatment with new RT technologies and ICIs.

## Simple Summary

Radiation therapy is highly effective for treating head and neck cancer. However, patients with more advanced cancer still have a high risk of recurrence. Patients can also experience substantial side effects during and after radiation therapy for head and neck cancer. Recent technological developments have improved the efficacy and reduced side effects from radiation. Some of these, especially intensity modulated radiotherapy, have become widely adopted. In this review, we highlight these advances and suggest the appropriate clinical scenarios for their application. Furthermore, we discuss the potential of combined treatment using radiation and systemic therapy, especially immunotherapy, to further improve patient outcomes. This review can help physicians in choosing the right radiation treatment for their patients and scientists in focusing their future research effort.

## 1. Introduction

Conventional RT delivered using 2D or 3D techniques for head and neck squamous cell carcinoma (HNSCC) is limited by a high rate of toxicities and frequent locoregional recurrences. Technological advances, mainly the advent of intensity-modulated radiotherapy (IMRT), in the past few decades have substantially reduced short- and long-term toxicities, while altered fractionation and systemic therapy dramatically improved the cure rate. Nevertheless, IMRT for HNSCC is still associated with significant short and long-term toxicities, and recurrences remain common, especially in non-HPV related cancers. New methods of IMRT planning have the potential to further reduce radiation-related toxicities and increase the cure rate. The use of newer conformal RT modalities, such as stereotactic body radiotherapy (SBRT) and proton therapy (PT), is increasing. Interest in spatially fractionated RT (SFRT), such as GRID and LATTICE, is also growing. However, further research is needed to clarify the normal tissue effects and refine their use in clinic. In recent years, immune checkpoint inhibitors (ICIs) have emerged as an important element in the treatment of recurrent and metastatic HNSCC. Research has flourished attempting to create synergy between RT and ICIs. In this review, we summarize the evolution of RT technologies for HNSCC, from altered fractionation to IMRT, PT, SBRT, and SFRT, focusing on the latest clinical evidence on tumor control and toxicities, and outline opportunities for the synergistic use of newer RT modalities and ICIs.

Search strategy: Primary references were selected from PubMed searches using terms for individual RT technologies (“altered fractionation”, “intensity modulated radiotherapy”, “proton therapy”, “stereotactic body radiotherapy”, or “spatially fractionated radiotherapy”) in combination with “head and neck cancer”. Relevant studies were selected as a representative sample of the literature, rather than a systematic review.

## 2. Altered Fractionation

Various altered fractionation schedules have been tested to exploit the 4Rs of radiobiology: repair, redistribution, re-oxygenation, and repopulation [1]. RTOG 9003 was a randomized controlled trial (RCT) comparing three altered fractionation schemes to standard 7-week fractionation: hyperfractionation with increased nominal dose, accelerated fractionation with split course, and accelerated fractionation with concomitant boost [2]. Hyperfractionation was found to be superior in both locoregional control (LRC) and overall survival (OS) than standard fractionation [3]. The first Meta-Analysis of Radiotherapy in Carcinomas of Head and neck (MARCH) of 15 randomized controlled trials (RCTs) showed improved OS with altered fractionation compared to standard fractionation (hazard ratio (HR) 0.92, *p* = 0.003). This benefit was mostly due to improvement in LRC (9.4% at 5 years) and OS (8.2% at 5 years) using hyperfractionation [4].

The enthusiasm for altered fractionation waned after concurrent chemotherapy was found to provide similar magnitude of benefit compared to altered fractionation alone (4% increase in OS at 5 years according to the MACH-NC meta-analysis) [5]. In the second MARCH meta-analysis published in 2017, including 19 new RCTs, standard fractionation with concurrent chemotherapy was found to be superior to altered fractionation alone (mainly moderate acceleration), with an absolute OS benefit of 5.8% at 5 years [6]. Even among patients receiving hyperfractionation, concurrent chemotherapy seemed to improve OS and LRC without an increase in acute or late toxicities [7].

In the presence of concurrent chemotherapy, the question of whether altered fractionation provides added benefit over standard fractionation was explored in several subsequent RCTs. RTOG 0129 randomized 743 patients with stage III or IV HNSCC to 70 Gy in 35 daily fractions over 7 weeks vs. 72 Gy in 42 fractions over 6 weeks (moderate acceleration using concomitant boost), both with concurrent high-dose cisplatin. No difference was observed in OS or LRC between the two arms [8]. Of note, IMRT was not allowed in RTOG 0129. A subsequent meta-analysis of four RCTs (including RTOG 0129) confirmed the lack of added benefit of moderate acceleration in the era of concurrent chemotherapy [9]. One unpublished trial (EORTC 22962) did suggest an improvement in LRC and OS comparing hyperfractionation vs. standard fractionation, both with chemotherapy [9]. However, details about this trial are scant, and it was conducted prior to the IMRT era (1996–1999).

In addition to chemotherapy, the arrival of IMRT has also hastened the diminution of interest in altered fractionation for several reasons. First, altered fractionation, by definition, requires changes in the standard treatment schedule, which can cause strain in personnel and resources and increase burden to patients [10]. Second, the benefit of hyperfractionation was mainly demonstrated using conventional RT, which is much more sensitive to fraction size given the large amount of normal tissue encompassed in the treatment field. IMRT on the other hand offers the capability of simultaneous boost to the tumor while minimizing fractional dose to the normal tissue, thus obviating the need to reduce fraction size. Nevertheless, hyperfractionation using IMRT is feasible and may be beneficial in certain circumstances. One single-arm study used simultaneous integrated boost (SIB) technique to treat the high, intermediate, and low risk volumes to a dose of 1.25, 1.07, and 0.89 Gy per fraction to a total dose of 70 Gy with concurrent cisplatin. Three-year LRC was 87%, and there was no failure in the elective volume [11]. More recently, an RCT in patients with nasopharyngeal carcinoma undergoing IMRT re-irradiation and concurrent chemotherapy demonstrated improved 3-year OS with hyperfractionation compared to standard fractionation (75 vs. 55%, *p* = 0.01). Interestingly, the OS benefit appeared to arise from reduction in RT-related toxicities and death (grade 5 late toxicity 7% vs. 24%) rather than increase in oncologic control (3-year LRC 60% in both arms) [12]. Therefore, judicious use of hyperfractionation may still yield clinically meaningful benefit with IMRT.

Altered fractionation is commonly used in the palliative setting for HNSCC. In particular, quad shot (QS) with 14–14.8 Gy in four fractions over two consecutive days is a widely used palliative regimen for HNSCC [13,14]. The QS regimen is well tolerated and can be repeated for up to 3–4 cycles with 2–4 weeks of interval between cycles [13]. Large retrospective series have demonstrated response rates up to 70–90% [15,16]. In a large series of 166 patients who received previous radiation, QS provided palliative response in 66%, and up to 86% among those who received 3–4 cycles. Moreover, highly conformal radiation techniques, such as IMRT and PT, may further improve the therapeutic ratio of QS [17,18]. Recently, reports have emerged that suggest an immunostimulatory effect of QS. Cases of pathologic complete response and even abscopal effect after QS have been reported [19,20]. In one retrospective study, patients who received concurrent ICIs had higher rate of local control at 12 months after QS (85 vs. 63%) [21]. Prospective trials, such as NCT04454489, are being conducted to further evaluate the potential synergy between QS and ICIs [22].

Chapter conclusion: Despite improved LRC and OS in the conventional RT era, altered fractionation, in particular, hyperfractionation, has not been shown to be beneficial in randomized controlled trials in the era of concurrent chemoradiotherapy and IMRT. Nevertheless, hyperfractionation may be advantageous in certain situations, such as reirradiation.

## 3. Intensity Modulated Radiotherapy

### 3.1. Normal Tissue Sparing

The ability of IMRT to spare organs at risk (OARs) in head and neck cancer have been repeatedly demonstrated. Table 1 summarizes the key RCTs comparing IMRT to conventional 2DRT or 3D-CRT in reducing xerostomia [23,24,25,26]. Also included are studies using dose-optimized IMRT for the sparing of xerostomia or dysphagia OARs [27,28]. Of note, most of these studies have a relatively short follow up (1 year) for the primary endpoint. However, durable efficacy has been confirmed in two RCTs after a median follow up of 15.5 and 11.7 years, respectively [29,30]. Based on these advantages, IMRT is recommended by most society guidelines as the preferred RT modality for the treatment of HNSCC [31].

Since its inception, IMRT techniques continue to evolve as our understanding of normal tissue injury improves. It has become clear that dose to the OARs can be further optimized beyond what has been achieved using traditional dose-volume approaches. Functional avoidance by preferentially sparing certain substructures, in addition to anatomic avoidance of the entire OAR, may be advantageous based on the biological and clinical observations that different substructures of OARs can respond differently to radiation. For example, the anterior-superior portion of the parotid gland that corresponds to the periductal stem cell-rich region is responsible for post-radiation saliva recovery and is more sensitive to low dose bath [32,33]. This has spurred prospective trials of selectively sparing this area of the parotid, including an ongoing RCT comparing MR sialography guided parotid duct sparing vs. standard IMRT (NCT06276946) [34,35].

How to optimally spare multiple OARs simultaneously is not straightforward, even with IMRT. Advances in computational techniques are moving us closer to the ideal of “optimal sparing” of all OARs. For example, geometry-based feasibility dose-volume histograms (fDVHs) of different OARs can provide guidance on the physically achievable dosimetry based on the patient’s own anatomy without a priori knowledge of beam arrangements [36]. This tool has been tested prospectively in patients receiving definitive RT for oropharynx cancer. Patients treated with fDVH-guided IMRT planning had less variability in the dose to the parotids and larynx, and reported less dry mouth, sticky saliva, and pain at 6 months post-RT [37]. Other methods to optimize IMRT planning, such as multi-criteria optimization and knowledge-based planning using artificial intelligence, may also be useful for this purpose [38,39,40].

### 3.2. Enhancing Tumor Control

One key feature that distinguishes IMRT from conventional RT is the ability to differentially dose certain areas of the target during the same fraction using “dose painting”, or simultaneous integrated boost (SIB) [41,42,43,44]. Dosimetrically, SIB is more convenient to plan and offers greater conformality with less low dose spread [45]. Planning studies have shown reduced dose to most OARs using SIB, including the brainstem, spinal cord, parotid and submandibular glands, and larynx, compared to sequential boost with the same target coverage [45,46]. Similar oncologic and toxicity outcomes were shown in an RCT comparing SIB to sequential boost techniques [47,48].

One potential advantage of SIB is dose escalation without prolongation of the total treatment time. Early planning studies demonstrated the feasibility of dose escalation using SIB to at least 86 Gy without compromising the brainstem or spinal cord [49]. In a phase I-II study, 57 patients received 69, 72, and 75 Gy over 30 fractions using SIB approach without concurrent chemotherapy. The rate of grade 3–4 mucositis was 26%, 42%, and 37%, respectively, similar to conventional chemoradiotherapy (CRT) [50]. There may still be an upper limit on fraction size, even with IMRT, as some patients in the early dose escalation studies developed dose limiting toxicities after 70.8–73.8 Gy in 30 fractions (2.36 and 2.46 Gy per fraction, respectively) [51]. Moreover, data are lacking on the long-term toxicities with SIB dose escalation.

Akin to the functional sparing of OARs, recent studies have focused on “functional targeting” of substructure of the tumor thought to be most clonogenic and radioresistant [52]. These efforts are supported by observations showing that recurrences often occur in areas of initial ^18^F-fluorodeoxyglucose (FDG) avidity and regions with lower apparent diffusion coefficients (ADC) on diffusion weighted MRI (DW-MRI) [53]. Table 2 summarizes dose escalation studies using ^18^F-FDG PET [54,55,56,57,58]. Doses up to 95.9 Gy in 32 fractions have been attempted with promising tumor control. However, late mucosal ulcer was often the dose limiting toxicity (23% grade 4 in one study). This seems to be associated with post-treatment exposure to smoking or alcohol, which is common in patients with HNSCC [57]. Therefore, functional dose escalation for HNC should be approached carefully on clinical trials.

Two RCTs have demonstrated encouraging results with DW-MRI guided dose escalation. One study randomized 260 patients with stage III-IVA nasopharyngeal carcinoma to standard CRT of 70.4–72.6 Gy vs. dose escalation to the ADC-low region of GTV to a dose of 75.2–77.55 Gy in 32–33 fractions. There was no difference in acute or late toxicities, but the dose escalation arm had better locoregional and distant control, as well as disease free survival [59]. In the second study, 81 patients with locally advanced HNSCC were randomized to DW-MRI guided dose escalated CRT vs. standard 70 Gy CRT over 35 fractions in the control arm. Patients in the experimental arm underwent multiparametric MRI at baseline and after 2 weeks, and those with persistent regions low in blood volume and ADC received 2.5 Gy per fraction for 20 fractions (total 80 Gy). Locoregional failure was reduced from 40% to 18% in the dose escalation arm. Acute and late toxicities, as well as patient reported outcomes, were similar between the arms [60]. However, both studies have relatively short follow up (median 25 and 29 months, respectively). Therefore, late toxicities, especially soft tissue fibrosis, dysphagia, trismus, and laryngeal complications, are not yet well understood with this approach.

### 3.3. IMRT vs. Brachytherapy

Brachytherapy benefits from the inverse square law in normal tissue sparing. Similar to IMRT, brachytherapy can achieve dose escalation while still respecting normal tissue tolerance. Interstitial or intracavitary brachytherapy has been used as the sole treatment or as a boost in combination with external beam radiotherapy for cancer of the oral cavity, oropharynx, and nasopharynx with high rates of local control [61,62,63]. Early studies also suggested reduction in radiation-related toxicities, such as dysphagia, using brachytherapy boost over conventional RT alone [64]. However, subsequent dosimetric and retrospective studies showed equivalent or better normal tissue sparing with IMRT [65,66]. Moreover, brachytherapy has several disadvantages, including invasiveness of interstitial implant, risk of soft tissue and bone necrosis, and requirement of special training and expertise. Access to brachytherapy for HNSCC is limited to specialized centers with prior experience. In the era of IMRT, brachytherapy may still have advantages in certain clinical scenarios, such as recurrence after prior radiation [67,68]. Updated recommendations for patient selection, implant techniques, planning, and dose regimens for head and neck cancer brachytherapy were recently published by GEC-ESTRO [69].

Chapter conclusion: In summary, IMRT has enabled a quantum leap in our ability to widen the therapeutic window for HNSCC. We have come to understand the functional significance of different substructures of OARs and the tumor. Ongoing research in selective sub-OAR sparing and sub-tumor dose escalation will hopefully yield further improvement in the definitive treatment of HNSCC.

## 4. Proton Therapy

**Figure 1:**
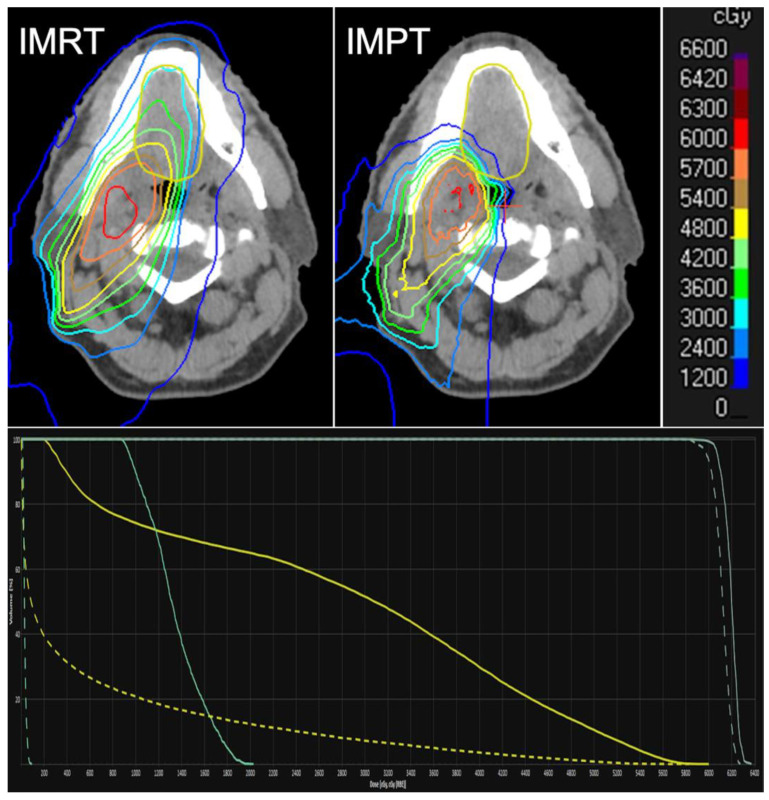
Top: A comparison of intensity modulated radiotherapy (IMRT) vs. intensity modulated proton therapy (IMPT) for a tonsillar squamous cell carcinoma. Bottom: Dose volume histogram of the oral cavity (yellow) and left submandibular gland (turquoise) from IMRT (solid lines) vs. IMPT (dashed lines).

In parallel to the proliferation of proton therapy (PT) centers in the United States and around the world, PT is increasingly utilized for patients with HNSCC. A recent survey conducted by the National Association for Proton Therapy showed 2303 patients with head and neck cancer treated with PT, a seven-fold increase from 2012 to 2021 [70]. The interest in PT is at least partly due to the unique physical properties of protons compared to X-ray. The dose deposition by protons is characterized by a sharp Bragg peak where the bulk of the dose is deposited followed by rapid fall off to zero within a few millimeters beyond the Bragg peak. Unlike photons, there is no exit dose with proton therapy, which can result in lower physical dose to the OARs and reduced the integral dose overall (Figure 1). It is hypothesized that this dosimetric advantage can translate into reduced short- and long-term toxicities compared to IMRT in head and neck cancer. In this section, we will take a nuanced look at this question, with the understanding that no level 1 evidence has been published to confirm this hypothesis.

Reduced doses to the spinal cord, brainstem, oral cavity, and salivary glands can be observed with PT in patients receiving unilateral and bilateral neck irradiation compared to IMRT [71,72]. This ability to spare the brainstem and spinal cord is particularly relevant in tumors of the skull base (sinonasal or nasopharynx) and in patients receiving high-dose re-irradiation. Patel et al. identified 41 observational studies of sinonasal tumors and concluded that particle radiotherapy is associated with improved outcomes including 5-year overall survival and disease-free survival (when compared with photons) [73]. Large cohorts of patients receiving PT re-irradiation have also been reported, with reasonable oncologic and toxicity outcomes [74,75,76].

Retrospective studies of the clinical outcomes using intensity-modulated PT (IMPT) have been reported. In a case-controlled study from MD Anderson Cancer Center, 50 patients with oropharynx cancer were treated with IMPT, compared to 100 treated with IMRT. There was a trend toward reduced gastrostomy tube dependence at 3 months [77]. In a similar study from Mayo Clinic, 305 patients with oropharynx cancer were treated with IMPT (N = 46) vs. IMRT (N = 259). Reduced narcotic use, acute mucositis, and feeding tube dependence were observed with IMPT [78]. Memorial Sloan Kettering Cancer Center also reported results from patients with oropharynx cancer treated with IMPT (N = 58) vs. IMRT (N-234). Both acute and late toxicities were reduced in the IMPT group: grade 2+ oral pain 72 vs. 93% (*p* < 0.001), grade 3+ mucositis 53 vs. 70% (*p* = 0.003), grade 2+ xerostomia 21 vs. 29% (*p* < 0.001), grade 2+ dysgeusia 28 vs. 57% (*p* < 0.001), grade 3 dysphagia 7 vs. 12% (*p* < 0.001) [79]. In all studies, OS and PFS appeared similar between IMPT and IMRT. A phase III, multi-center, non-inferiority RCT (NCT01893307) is being conducted to compare IMPT and IMRT in patients with oropharyngeal carcinoma. After 440 patients have been randomized with a median follow up of 3.14 years, the primary endpoint of non-inferior PFS at 3 years for IMPT was met (hazard ratio 0.87, 95% confidence interval 0.56–1.35) [80]. Encouragingly, gastrotomy tube dependence was decreased in the IMPT arm compared to IMRT (42% vs. 28%, *p* = 0.019). Formal publication of these results is eagerly awaited.

There are also strong data suggesting a decreased risk of secondary malignancies with PT. One such pivotal study published by Chung et al. in 2013 reviewed data for >1000 patients and found a 2.3% decrease in the risk of second malignancies (5.2 vs. 7.5%) with patients undergoing PT when compared with photons [81]. More recently, Xiang et al. in 2020 reviewed data from >450,000 patients in the National Cancer Center Database, 1.3% of whom received PT and 65.2% IMRT. PT was associated with a significantly lower risk of second cancers for primary tumors of the head and neck (adjusted OR 0.42, 95% CI 0.22–0.81, *p* = 0.009) [82]. As a result, PT is often favored in younger patients for whom secondary malignancies from radiation is of greater concern.

Paradoxically, increased biological dose, and sometimes injury, to the mucosa and mandible from PT have been reported. In a planning study of IMPT vs. IMRT for recurrent nasopharyngeal carcinoma (NPC), lower maximum doses to the spinal cord, brainstem, and optic nerves were observed with IMPT than IMRT. However, high non-uniformity was observed within the target, resulting in significantly higher dose to the internal carotid artery (68 Gy[RBE] vs. 62.6 Gy, *p* < 0.01) and (72 Gy[RBE] vs. 62.8 Gy, *p* < 0.01) [83]. In a murine model, PT resulted in higher rate of mucositis compared to X-ray irradiation [84]. Clinically, increased uptake on ^18^F-FDG PET can be observed within the target volume after IMPT, leading to a higher estimated relative biological effectiveness (RBE) of 1.2–1.5 than the commonly used RBE of 1.1 [85].

The RBE of PT for mandibular osteoradionecrosis (ORN) may also be higher than previously estimated. In a large cohort of patients treated with volumetric modulated arc therapy (VMAT) vs. IMPT, the volume of mandible receiving 40–60 Gy (assuming RBE of 1.1) was significantly lower with IMPT: V40Gy[RBE] 21 vs. 39 cc, V50Gy[RBE] 16 vs. 32 cc, V60Gy[RBE] 4 vs. 18 cc. These were similar to the dose levels identified in patients with ORN after treatment with 3D and IMRT techniques [86]. However, the rate of ORN between the two groups were similar (9 of 335 IMPT vs. 8 of 335 matched VMAT controls). This resulted in an estimated RBE of 1.24–1.58 for IMPT doses of 40–60 Gy (lower with higher proton dose) [87]. Similarly, another case series reported a rate of 10.6% for mandibular ORN after IMPT, close to the expected rate of ORN after IMRT (not directly compared in this study) [88].

Perhaps the most intriguing prospect of the reduced integral dose from IMPT is the potential decrease in immune suppression, which may translate into more synergy with ICIs. Severe lymphopenia can result from radiation treatment and is associated with increased distant metastasis and decreased OS [89]. Reducing the low dose bath (10–25 Gy) to the bone marrow and other large organs, such as the brain and lungs, may decrease severe lymphopenia [90]. A recent RCT has demonstrated reduced incidence of severe lymphopenia in patients receiving PT for esophageal cancer compared to IMRT [91]. Although data is not yet available, a single-arm, prospective trial (NCT05364411) has been launched to further investigate the impact of PT on the immune system in patients with HNSCC [92]. Combined use of PT and ICIs is being investigated, and early results showed an encouraging objective response rate of 23% in a previously treated cohort of patients with recurrent or metastatic HNSCC [93].

Chapter conclusion: In summary, PT has the dosimetric advantage of no exit dose and lower integral dose compared to IMRT. There are strong data supporting the utility of PT in younger patients, those with skull base tumors, and those receiving high-dose re-irradiation. Retrospective case series suggest that PT may reduce dysphagia and xerostomia, but the RBE for some OARs, including the oropharyngeal mucosa and mandible, may be higher than commonly assumed. RCTs comparing IMPT and IMRT are underway, including NCT01893307 in the United States, ISRCTN16424014 in the United Kingdom, and NCT04607694 in Denmark, but data so far are limited to retrospective series. Lastly, prospective trials may help confirm the reduction in lymphopenia and immune suppression with PT, which may enhance the synergy between RT and ICIs.

## 5. Stereotactic Body Radiotherapy (SBRT)

In contrast to conventional RT or IMRT, which aims to deliver a homogeneous dose within the target, SBRT, or stereotactic ablative radiotherapy (SABR), typically delivers a highly inhomogeneous dose within the target, with hotpots typically up to 120–160%, and sharp dose fall off outside the target. It also delivers a higher dose, typically 6–10 Gy, per fraction. SBRT causes distinct radiobiological processes beyond direct cell killing to enhance tumor control [94]. First, SBRT can ablate tumor vasculature leading to indirect tumor death [95,96]. Second, fractionated SBRT can also improve re-oxygenation in poorly vascularized, hypoxic tumors [97]. Lastly, conventionally fractionated EBRT can cause lymphopenia and suppress anti-tumor immunity [98]. In contrast, SBRT can induce expression of tumor neoantigen and enhance anti-tumor immunity [99]. A recent preclinical study has demonstrated the importance of dose heterogeneity in promoting an anti-tumor transformation of the tumor microenvironment, including an increase in effector T cell cytokines and clonal expansion of effector CD8 T cells [100]. How to exploit these novel biological mechanisms, especially in combination with ICIs, is an active area of research.

**Figure 2:**
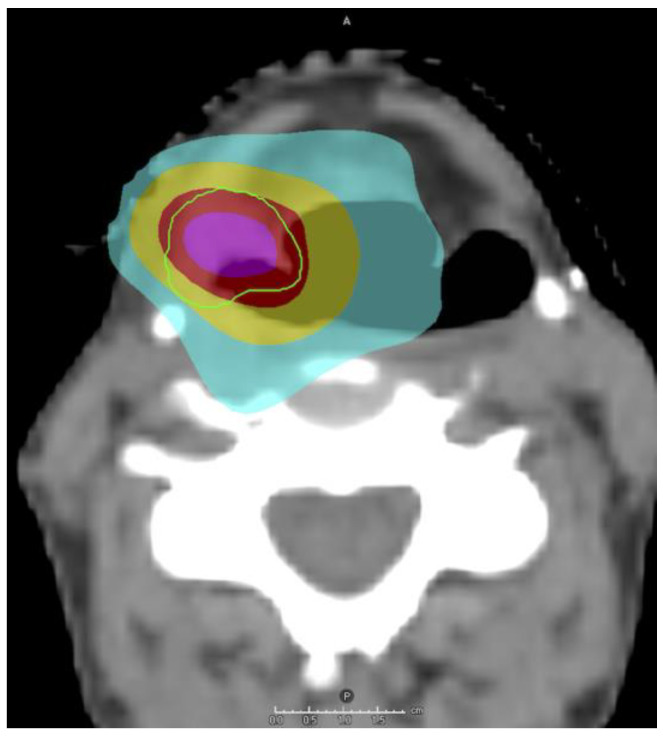
A stereotactic body radiotherapy (SBRT) plan for a squamous cell carcinoma (SCC) of the right pyriform sinus in a patient who had undergone full-dose chemoradiotherapy for a base of tongue SCC eight years prior. Note the undercoverage of the posterolateral aspect of the target to respect dose constraints for the carotid artery. Green contour: planning target volume. Red color wash: prescription 35 Gy in five fractions. Purple color wash, 40 Gy; yellow color wash, 25 Gy; cyan color wash, 15 Gy. A, anterior; P, posterior.

Clinically, SBRT for HNSCC is often used in the recurrent or metastatic setting [101]. Figure 2 shows an SBRT plan for a patient with a new pyriform sinus SCC 8 years after undergoing CRT for a left base of tongue SCC. A systematic review and meta-analysis of 10 studies involving 575 patients with previously irradiated, recurrent HNSCC treated with SBRT showed 2-year local control of 47% and overall survival (OS) of 30% [102]. Tumor burden and total dose are the main driver of outcomes. A multi-institution cohort of 414 patients treated with IMRT vs. SBRT reirradiation demonstrated similar OS with IMRT and higher-dose SBRT (≥35 Gy) for patients with small tumors (≤25 cc), while those treated with lower dose (<35 Gy) or with larger tumors (>25 cc) had worse OS with SBRT [103]. Severe SBRT-related toxicities were infrequent (9.6% grade 3), however, grade 5 toxicities have been reported in 4.6% of patients [102]. Both acute and late toxicities may be enhanced due to the high dose per fraction and shortened total treatment time. These include pharyngeal ulcer, laryngeal chondronecrosis, brachial plexopathy, and carotid blow out [104,105]. SBRT for mucosal sites, especially laryngeal and hypopharyngeal areas, are associated with higher rates of both acute and late toxicities compared to SBRT for nodal disease [105]. Normal tissue complication probability (NTCP) analyses for these toxicities have been published by multiple groups [106,107,108].

To improve patient outcomes with recurrent HNC, recent studies have focused on the combined application of SBRT with systemic agents. Cetuximab, an anti-epidermal growth factor receptor (EGFR) antibody, has been studied most in this setting. Two studies of 110 patients with recurrent HNSCC treated with SBRT and concurrent cetuximab have shown similar grade 3 toxicity of 6–9% and median PFS of 7 months [109,110]. A more recent study of 74 previously irradiated patients treated with SBRT and cetuximab showed median PFS of 9 months. However, late grade 3 or higher toxicities occurred in 20%, including five ORN or soft tissue necroses and one carotid blow out [111]. Large RCTs, including RTOG 1016, De-ESCALaTE HPV, and TROG 12.01, comparing cetuximab to cisplatin have demonstrated superior tumor control with cisplatin, even in patients with low-risk HPV-related oropharyngeal carcinoma, and similar rate of severe toxicities [112,113,114]. A recent phase I trial has explored the used of concurrent cisplatin with SBRT. Eighteen patients received 5-fraction SBRT with cisplatin given at 15 mg/m^2^ before every fraction. No dose limiting toxicity was observed up to 40 Gy [115].

Harnessing the immune modulatory effect of SBRT in combination with ICIs is under intense research for HNSCC. In the neoadjuvant setting, SBRT 24 Gy in three fractions and durvalumab, an anti-PD-L1 inhibitor, produced a major pathologic response rate of 89% in patients with oral cavity SCC. There was a signal of immune stimulation as evidenced by increased circulating effector T cells, decreased immunosuppressive cells, and increased neoantigen presentation after SBRT [116]. However, a recent RCT compared nivolumab, an anti-PD-1 inhibitor, with and without SBRT in patients with metastatic HNSCC. No improvement in OS, PFS or response duration was observed, and there was no evidence of an abscopal effect [117]. Further studies are clearly needed to optimize the immune stimulatory effect of SBRT in the presence of ICIs. For example, a phase II study (NCT04862455) is testing whether an intratumoral injection of a radiosensitizing hafnium oxide nanoparticle prior to SBRT could enhance the activity of pembrolizumab.

In the upfront setting, SBRT may be useful in certain situations as well. In previously untreated elderly patients who were not candidates for definitive surgery or radiotherapy, SBRT provided a local control of 69% and 86% at 1 year in two series of 60 patients in total, with a low rate of grade 3 or higher toxicities [118,119]. There may also be a role for SBRT in patients with early glottic cancer. In one trial, 12 patients received 42.5 Gy in five fractions for Tis to T2 glottic cancer, and none had local recurrence after median followed up of 39 months [120]. However, a similar trial using slightly higher dose and more protracted schedule (59.5 Gy in 17 fractions or 55 Gy in 11 fractions) yielded unacceptable toxicities, including two grade 3 laryngeal inflammations, one causing arytenoid chondronecrosis requiring supraglottic laryngectomy [121]. The substantially shortened total treatment time in SBRT may be the reason for the accentuated mucosal toxicity observed in some series.

Chapter conclusion: In summary, the utility of SBRT is not clearly defined currently for the lack of RCTs comparing SBRT to other RT modalities. In the recurrent and metastatic setting, some institutions prefer SBRT for its shortened course and sharp dose fall off. However, severe toxicities, both acute and late, are possible. More research is needed to define the optimal patient selection criteria and dose and fractionation schemes for SBRT in HNSCC. Synergistic use of SBRT and ICIs is of great interest to the field, although clinical benefit is yet to be demonstrated.

## 6. Spatially Fractionated Radiotherapy

SFRT, including GRID and LATTICE RT, is another form of highly inhomogeneous RT technique. The concept of spatial fractionation was initially introduced in the early 1900s due to the limitation of high surface dose from low-energy X-rays when treating deeply seated tumors [122,123]. A metal grid was used to deliver high doses through small, interspersed openings and allow recovery of the surrounding skin through sparing of stem cells in the blocked areas [124]. However, the advent of skin-sparing megavoltage X-rays mitigated the need for GRID therapy until recently when new biologic mechanisms and clinical utility of SFRT were recognized [125]. The biologic effects of SFRT may be similar to SBRT, where the inhomogeneous dose distribution can normalize aberrant tumor vasculature, overcome tumor hypoxia, and enhance anti-tumor immune response [126,127,128]. Dramatic responses to combined SFRT and ICIs have been reported [129,130].

**Figure 3:**
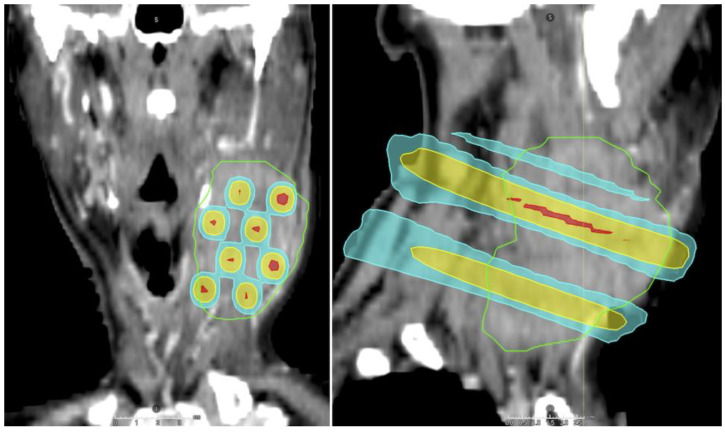
A multi-leaf collimator-based GRID radiotherapy plan for a bulky, unresectable left neck node from an oral cavity squamous cell carcinoma. A course of palliative radiotherapy was delivered subsequently after GRID RT due to patient’s advanced age and concurrent medical comorbidities. Green contour: planning target volume. Red color wash: prescription to the peak 10 Gy in 1 fraction. Yellow color wash, 5 Gy; cyan color wash, 2.5 Gy. S, superior; I, inferior.

Modern GRID RT uses a sieve-like metal block or multi-leaf collimators (MLCs, Figure 3) to generate high-dose (peak) and intervening low-dose (valley) areas throughout the tumor. In contrast, LATTICE RT creates small (typically 1 cm in diameter) islands of high-dose spheres (vertices) using conformal techniques, such as VMAT [131]. A typical GRID or LATTICE treatment prescribes 15–20 Gy in one fraction to the peaks or vertices. SFRT can be used as standalone treatment for palliation or in conjunction with a course of conventional or hypofractionated RT for palliative or definitive treatment.

Data on the clinical application of SFRT in HNSCC are limited to small, single-institution case series. In the palliative setting, clinical response to GRID followed by EBRT can be seen in 70–80% of patients treated for pain and mass effect [132,133]. In fact, patients with HNSCC have higher response rate to GRID RT compared to those with cancer in other sites [132]. After LATTICE RT of 15 Gy (followed by conventional RT of 30 Gy in 10 fractions), complete response has been observed in a patient with a bulky axillary metastasis from skin SCC not suitable for SBRT [134]. A multi-center study of LATTICE RT for palliation in 30 patients (only four to the head and neck) showed 100% symptom relief within 1–8 days and an 89% overall clinical response rate. No grade 3 or higher toxicities were observed [135]. There are also limited data supporting SFRT for the definitive treatment of HNSCC. In a series of 14 patients, most with N3 disease, treated with definitive GRID RT followed by conventional RT to a median of 70 Gy, the rate of neck control was 93% at a median follow up of 10 months. In the same series, 13 patients received preoperative GRID, and the rate of pathologic CR was 85% [136]. Another series reported 14 patients with bulky (≥6 cm) HNSCC who received GRID RT followed by definitive chemoradiotherapy with IMRT. Six of the 14 patients received GRID to the primary tumor, while the rest received it to the neck. At a median follow up of 20 months, control of the GRID sites was 79% [137].

Future clinical development of SFRT for HNSCC faces several hurdles [138]. First, SFRT planning is subjective, as there is no consensus on the optimal dose and spatial placement of the peaks and vertices. Second, a new radiobiological framework is needed to describe treatment effects from a highly inhomogeneous field like SBRT and SFRT. Traditional tumor control probability (TCP) and NTCP analyses are inadequate in this regard. The concept of equivalent uniform dose (EUD) as proposed by Niemierko may be useful in this aspect [139,140]. Lastly, toxicities of SFRT must be better defined and reported, especially in combination with EBRT and systemic therapy. International consensus recommendations on patient selection, treatment planning, post-treatment assessment and translational endpoints have been published to guide the design of future trials using SFRT [138,141]. Based on these recommendations, the suggested SFRT dose for HNSCC in the definitive setting is 15 Gy in one fraction and GRID technique is favored over LATTICE due to the lack of published clinical experience with the latter [141].

Chapter conclusion: There is renewed interest in SFRT for advanced HNSCC. Challenges and consensus guidelines for future trials using SFRT have been published.

## 7. Discussion

RT techniques for HNSCC have evolved from the original opposed lateral fields to highly conformal and sometimes inhomogeneous dose delivery with attention to specific areas of the targets and OARs. There are randomized and population level data that demonstrate significant therapeutic gain with IMRT compared to conventional RT [142,143]. However, many patients with locally advanced HNSCC still recur after radical CRT, and most patients with recurrent or metastatic HNSCC die of their disease. Moreover, RT-related toxicities remain high even in the era of IMRT. We must advance the field of radiation oncology to meet the needs of our patients by developing new RT techniques to improve tumor control and reduce toxicities.

To this end, several lines of research are needed. First, RT dose escalation may be feasible and beneficial in certain patients with biomarkers defined on functional imaging [59,60]. Future dose escalation studies should focus on refining the optimal dose and fractionation required for improved tumor control. Imaging biomarkers used in these studies are not routinely obtained for patients with HNSCC. Whether DW-MRI or PET-based biomarkers can be obtained consistently in a broader patient population remains to be seen. Lastly, toxicities from dose escalation studies should be carefully documented and reported for future analysis on normal tissue toxicities.

To improve sparing of OARs, techniques such as selective substructure avoidance or computational tools such as geometry-based dose prediction have proven useful [35,37]. Questions remain how effective these tools are clinically. Patient reported outcomes need to be collected prospectively to better understand the clinical benefit with these highly specialized treatments [144]. Moreover, the underlying biology of radiation injuries is poorly understood. Basic and translational research using animal models and novel imaging modalities will hopefully lead to identification of novel biomarkers for normal tissue injury beyond simple dosimetric parameters.

Lastly, just as concurrent chemotherapy with IMRT has revolutionized the care for HNSCC in the last two decades, how to best integrate novel RT techniques, such as IMPT, SBRT and SFRT, with novel systemic therapies, such as ICIs, remains to be determined [117]. In this area, greater understanding of the immune microenvironment in HNSCC and how it interacts with different RT modalities will be critical [116]. Our current preclinical models are inadequate to recapitulate the highly conformal and often inhomogeneous RT modalities such as SBRT and SFRT. New preclinical models are needed, along with systematic identification and validation of immune biomarkers in clinical trials, such as HYDRA [92]. Serum and immune biomarkers are likely critical in refining patient selection criteria for combined modality treatment. With these lines of inquiry, we may see a renaissance of combined modality therapy with novel RT techniques and ICIs for HNSCC in the coming years.

## Figures and Tables

**Table 1:** Key randomized controlled trials demonstrating sparing of xerostomia and dysphagia related organs using IMRT.

	Primary Sites	Treatment Arms	Chemotherapy	Bilateral RT	Primary Endpoint	Primary Outcomes	Secondary Outcomes
Kam et al., 2007 [23]	Nasopharynx (100%)	2DRT (*N* = 28) IMRT (*N* = 28)	None	68%	Observer-rated severe xerostomia at 1 year	82% vs. 39% (*p* = 0.001)	Fractional stimulated parotid flow rate 0.90 vs. 0.05 (*p* < 0.001) Fractional stimulated whole saliva flow rate 0.41 vs. 0.20 (*p* = 0.001)
Nutting et al., 2011 [24]	Oropharynx (85%) Hypopharynx (15%)	2DRT (*N* = 47) IMRT (*N* = 47)	None	N/A	Grade 2 or higher xerostomia at 1 year	74% vs. 38% (*p* = 0.003)	Fatigue 41% vs. 74% (*p* = 0.002, IMRT higher) Grade 2 or higher xerostomia at 2 years 83% vs. 29% (*p* < 0.001)
Gupta et al., 2012 [25]	Oropharynx (53%) Hypopharynx (28%) Larynx (19%)	3D-CRT (*N* = 28) IMRT (*N* = 32)	94%	N/A	Acute grade 2 or higher salivary toxicity	89% vs. 59% (*p* = 0.009)	Late xerostomia 6–36 months Late subcutaneous fibrosis 6–36 months Both significantly improved by IMRT
Tao et al., 2020 [26]	Oropharynx (85%) Oral cavity (3%) Hypopharynx (12%)	3D CRT (*N* = 94) IMRT (*N* = 94)	100%	N/A	Locoregional progression	27.7% vs. 23.5% at 3 years (NS)	Grade 2 or higher xerostomia at 1 year 63% vs. 23% (*p* < 0.001)
Huang et al., 2022 [27]	Nasopharynx (100%)	Standard IMRT (*N* = 45) Superficial parotid sparing IMRT (*N* = 45)	90%	100%	Any xerostomia at 1 year	95% vs. 83% (*p* = 0.007)	Unstimulated saliva flow rate at 1 year 0.35 vs. 0.67 (*p* = 0.02) Stimulated saliva flow rate at 1 year 0.32 vs. 0.66 (*p* = 0.02)
Nutting et al., 2023 [28]	Oropharynx (97%) Hypopharynx (3%)	Standard IMRT (*N* = 56) Dysphagia-optimized IMRT (*N* = 56)	86%	100%	MDADI composite score at 1 year	70.6 vs. 77.7 (*p* = 0.04, higher better)	Similar grade 3–4 acute and later toxicities

MDADI: MD Anderson Dysphagia Inventory. NS: Not significant.

**Table 2:** Prospective studies of ^18^F-FDG PET guided dose escalation using simultaneous integrated boost. LR, local recurrence; RR regional recurrence.

	*N*	Primary Sites	Dose Levels	Chemotherapy	Median Follow Up	Tumor Control	Acute Toxicity	Late Toxicity
Madani et al., 2007 [54]	41	39% Oropharynx 34% Hypopharynx 27% Larynx 2% Oral cavity	72.5 Gy (*N* = 23), 77.5 Gy (N = 18) in 32 fractions	56%	14 months	7 (17%) LR at median of 7 months	57% grade 3+ dysphagia (1 grade 4) 44% grade 3+ dermatitis (1 grade 4)	13% grade 3 dysphagia 3% ulcer
Madani et al., 2011 [55]	21	52% Oropharynx 19% Hypopharynx 19% Larynx 10% Oral cavity	80.9 Gy (*N* = 7), 85.9 Gy (N = 14) in 32 fractions	43%	25 months	5% LR, 7% RR at 2 years	None grade 4	6 (29%) mucosal ulcer at median of 7 months (5 at level 2)
Rasmussen et al., 2016 [56]	15	53% Oropharynx 13% Hypopharynx 33% Larynx	82 Gy in 34 fractions	100%	18 months	No in-field failure	4 (27%) mucosal ulcer, none grade 4	1 (7%) grade 4 (laryngeal dysfunction)
Olteanu et al., 2018 [57]	39	54% Oropharynx 18% Hypopharynx 20% Larynx 8% Oral cavity	66–95.9 Gy in 32 fractions	46%	37 months	2 (5%) LR, 3 (8%) RR	N/A	20 (51%) grade 1–3, 9 (23%) grade 4 mucosal ulcer at 3–10.5 months
Michaelidou et al., 2021 [58]	29	100% Oropharynx	71.5 Gy in 30 fractions	100%	36 months	4 (14%) LR, 2 (7%) RR	45% grade 3 dysphagia 3% grade 3 dermatitis, none grade 4	3 (10%) grade 3 dysphagia, 1 (3%) grade 3 mucosal atrophy
